# Meaningful Human Control over Autonomous Systems: A Philosophical Account

**DOI:** 10.3389/frobt.2018.00015

**Published:** 2018-02-28

**Authors:** Filippo Santoni de Sio, Jeroen van den Hoven

**Affiliations:** ^1^Section Ethics/Philosophy of Technology, Faculty Technology Policy and Management, Delft University of Technology, Delft, Netherlands

**Keywords:** meaningful human control, autonomous weapon systems, responsibility gap, ethics of robotics, responsible innovation in robotics, value-sensitive design in robotics, AI ethics, ethics of autonomous systems

## Abstract

Debates on lethal autonomous weapon systems have proliferated in the past 5 years. Ethical concerns have been voiced about a possible raise in the number of wrongs and crimes in military operations and about the creation of a “responsibility gap” for harms caused by these systems. To address these concerns, the principle of “meaningful human control” has been introduced in the legal–political debate; according to this principle, humans not computers and their algorithms should ultimately remain in control of, and thus morally responsible for, relevant decisions about (lethal) military operations. However, policy-makers and technical designers lack a detailed theory of what “meaningful human control” exactly means. In this paper, we lay the foundation of a philosophical account of meaningful human control, based on the concept of “guidance control” as elaborated in the philosophical debate on free will and moral responsibility. Following the ideals of “Responsible Innovation” and “Value-sensitive Design,” our account of meaningful human control is cast in the form of design requirements. We identify two general necessary conditions to be satisfied for an autonomous system to remain under meaningful human control: first, a “tracking” condition, according to which the system should be able to respond to both the relevant moral reasons of the humans designing and deploying the system and the relevant facts in the environment in which the system operates; second, a “tracing” condition, according to which the system should be designed in such a way as to grant the possibility to always trace back the outcome of its operations to at least one human along the chain of design and operation. As we think that meaningful human control can be one of the central notions in ethics of robotics and AI, in the last part of the paper, we start exploring the implications of our account for the design and use of non-military autonomous systems, for instance, self-driving cars.

## Introduction

Debates on lethal autonomous weapon systems have proliferated in the past 5 years. As a result of rapid and impressive developments in sensor technology, AI and machine learning, robotics and mechanical engineering, mechatronics, and systems with various degrees of autonomy will be available on a large scale in the coming years. These autonomous and semiautonomous systems are able to achieve goals and perform tasks without much intervention and control by human beings. A key question regarding both military and civil applications of these systems concerns responsibility for the consequences of their deployment. How can unacceptable risks be avoided, and how can human beings still be held responsible if systems are acting on their own accord, without little human control and intervention? What if armed drones, after being programmed and activated, could select and engage targets without further human intervention and civilians are mistakenly killed in an attack? What if—as happened in 2016—a driver of a car in autonomous mode is killed in a crash, because of the fact that a large white truck in front of the car is misclassified by the system as piece of the sky?

In this paper, we provide an analysis of the sort of control humans need to have over (semi)autonomous systems such that unreasonable risks are avoided, that human responsibility will not evaporate, and that is there is a place to turn to in case of untoward outcomes. We argue that higher levels of autonomy of systems can and should be combined with human control and responsibility. We apply the notion of *guidance control* that has been developed by Fischer and Ravizza (1998) in the philosophical debate about moral responsibility and free will, and we adapt it as to cover actions mediated by the use of (semi)autonomous robotic systems. As we will show, this analysis can be fruitfully applied in the context of autonomous weapon systems as well as of autonomous systems more generally. We think we herewith provide a first full-fledged philosophical account of “meaningful human control over autonomous systems.”

This paper is organized as follows. We first briefly review the existing literature on meaningful human control over autonomous weapon systems, and we identify three related issues to be addressed by a theory of meaningful human control (see Autonomous Systems and the Problem of Meaningful Human Control and Toward a Theory of Meaningful Human Control). We then briefly introduce the distinction between incompatibilist and compatibilist theories of moral responsibility, and we explain why we consider the compatibilist approaches to moral responsibility most suitable to ground a theory of meaningful human control over autonomous weapon systems (see The Philosophical Landscape: Control and Moral Responsibility). We introduce Fischer and Ravizza’s account of guidance control (see Conditions for “Guidance Control”). We expand, integrate, and translate it into a theory of meaningful human control over actions mediated by autonomous (weapon) systems and a set of design requirements to achieve this kind of control (see Meaningful Human Control: Tracking and Tracing Conditions and Meaningful Human Control over Autonomous Weapon Systems: Implications of Tracking and Tracing). Finally, we explain how our analysis of meaningful human control can be used outside the military field, and we pave the way for future work (see The Broader Picture: Meaningful Human Control and Responsible Innovation in Robotics).

## Autonomous Systems and the Problem of Meaningful Human Control

Autonomous weapon systems are “robot weapons that once launched will select and engage targets without further human intervention” (Altmann et al., 2013: 73).^1^ Britain, Israel, and Norway are already deploying autonomous weapon systems (Markoff, 2014)^2^ and it is long expected that other states will increasingly seek them (Singer, 2009).^3^ That this is not an unwarranted assumption may be clear from the fact that the high contracting parties to the UN have made this a central topic of debates and meetings of experts on autonomous technology and international humanitarian law at the UN Convention on Conventional Weapons and UNIDIR.^4^ Science and civil society have also addressed the issue. The prospect of a proliferation of autonomous weapon systems has created societal alarm, which issued in an international campaign for the ban of future fully autonomous weapon systems organized by NGO workers and academic scholars (stopkillerrobots.org), and an open letter signed by influential figures such as the physicist Stephen Hawking, Apple cofounder Steve Wozniak, and Tesla’s Elon Musk along with 1,000 AI and robotics researchers calling for a ban on “offensive autonomous weapons beyond meaningful human control” (Future of Life Institute, 2015). Scientists, entrepreneurs, policy-makers, and NGO workers involved in these initiatives agree that in order to prevent future robots from negatively impacting human society, we need to immediately start a systematic reflection on the ethical principles for the regulation and design of autonomous weapon systems (Russell et al., 2015). In august 2017, Elon Musk and 116 CEOs of tech companies drew attention to the dual-use issues of Artificial Intelligence and asked for a ban on lethal autonomous weapon systems (Gibbs, 2017).

In public and academic debate, autonomous weapon systems have been exposed to three main ethical objections: (a) as a matter of fact, robots of the near future will not be capable of making the sophisticated practical and moral distinctions required by the laws of armed conflict (Burridge, 2003; Sharkey, 2007, 2012; Asaro, 2008; Krishnan, 2009; Guarini and Bello, 2012): distinction between combatants and non-combatants, proportionality in the use of force, and military necessity of violent action. The delegation of military tasks to robots may therefore raise the number of wrongs and crimes in military operations (Sharkey, 2011). (b) As a matter of principle, it is morally wrong to let a machine be in control of the life and death of a human being, no matter how technologically advanced the machine is (Wagner, 2014). According to this position, which has been stated among others by The Holy See (Tomasi, 2013), these applications are *mala in se* (Wallach, 2013). (c) In the case of war crimes or fatal accidents, the presence of an autonomous weapon system in the operation may make it more difficult, or impossible altogether, to hold military personnel morally and legally responsible [the so-called responsibility gap problem: Matthias (2004) and Heyns (2013)].

In the legal–political debate on autonomous weapon systems of the past few years, these ethical concerns have been synthesized in the following principle:

*Principle* of meaningful human control future weapons systems must preserve meaningful human control over the use of (lethal) force, that is: humans not computers and their algorithms should ultimately remain in control of, and thus morally responsible for relevant decisions about (lethal) military operations. (Article 36, 2015)

This principle has attracted a wide consensus among scholars and policy-makers (Knuckey, 2014; Article 36, 2014; Horowitz and Scharre, 2015; Ekelhof, 2017), as “it offers more precision (control versus the somewhat ambiguous conceptual “loop” or the more passive “judgment”), it explicitly emphasizes the quality of control (“meaningful”), and it implicitly accords responsibility to human agents for decisions concerning each individual attack” (Vignard, 2014: 3).

Human Rights Watch, in an overview of the positions of different states on the matter, summarizes as follows^5^:

… the ICRC [International Committee of the Red Cross] concluded that “there appears to be broad agreement among States on the need to retain human control over the critical functions of weapon systems.” Colombia, for example, stated that “multilateral regulation is required” to ensure human control over deployed weapons. Croatia said, “[A]n international prohibition of weapons systems operating without meaningful human control should not be something unthinkable, particularly given the calls for a moratorium.” Denmark said that “[a]ll use of force must remain under meaningful human control.” Although not all states embraced the concept of meaningful human control, by November 2015 a total of nine states had called for a preemptive ban on fully autonomous weapons, which amounts to a requirement of meaningful human control over the use of weapons.

However, many scholars and parties to the debate have also recognized a serious *theoretical and practical problem* with the principle of meaningful human control:

*Problem* of meaningful human control policy-makers and technical designers lack a detailed theory of what “meaningful human control” exactly *means*; and therefore they don’t know which specific *legal regulations* and *design guidelines* should be derived from this principle. (Vignard, 2014; Horowitz and Scharre, 2015; Roff and Moyes, 2016)

## Toward a Theory of Meaningful Human Control

In this paper, we address the problem of meaningful human control, by laying the foundation of a philosophical account of this idea. We agree with David Mindell when he remarks that we need to move away from the myths and dreams about full autonomy and look at situated autonomy in real systems in the twenty-first century (Mindell, 2015: 10), and that we need to update our notions of “control” in order to come up with an actionable analysis of control in the age of smart machines. Our account of meaningful human control is based on insights from the literature on free will and moral responsibility, in particular the concept of “guidance control” as elaborated by Fischer and Ravizza (1998). While the starting point of this analysis is the concept of meaningful human control over autonomous weapon systems, we have the ambition to elaborate an account which can be applied to a broader range of autonomous systems, for instance, autonomous driving systems.

A second goal of the paper is to lay the foundation of a theory of meaningful human control that not only accommodates all relevant moral considerations, but is also suitable to give ethical guidance to policy-makers, engineers, and technical designers. We rely on the concepts of “Responsible Innovation” and “Value-sensitive Design” or “Design for Values” approach (Friedman and Kahn, 2003; van den Hoven, 2007, 2013), according to which in order to have a real impact on society, ethical constraints and aims should, first, shape technology in the *design* phase, where they still can make a difference, instead of fueling political and academic discussions when technology is already in place; and second, should eventually be cast in terms that can actually be designed for, i.e., in the form of requirements for systems, engineering, and software design.

Our account starts filling three gaps in the academic literature. Firstly, we offer an analysis of the notion of “control” that is based on the philosophical literature on free will and moral responsibility where the notion of *control* figures prominently. Computer and robot ethics researchers have already addressed the question, to what extent humans can and should remain morally responsible for the behavior of new sophisticated kinds of intelligent automated systems: military robots (Strawser, 2013; Di Nucci and Santoni de Sio, 2016; Galliott, 2016; Leveringhaus, 2016) but also computers (Bechtel, 1985; Friedman, 1990; Ladd, 1991; Nissenbaum, 1994; Kuflik, 1999; Johnson and Powers, 2005; Noorman, 2014), future “self-driving” cars (Maurer et al., 2015; Santoni de Sio, 2016), and healthcare robots (van Wynsberghe, 2015). However, they have not yet utilized the insights gained on the notion of control as it has been developed in philosophical theories of moral responsibility.

Secondly and relatedly, this paper contributes to the so-called compatibilist theory of moral responsibility which hold that individuals can be held responsible even if their actions can be explained in causal, possibly deterministic, terms. Existing compatibilist theories of moral responsibility have defined the conditions for morally relevant human control over everyday action, and they have addressed the challenges to moral and legal responsibility coming from recent progress in neuro- and social science (Dennett, 1984, 2004; Morse, 1994; Fischer and Ravizza, 1998; Vincent, 2013). Existing compatibilist theories, however, have not yet defined the conditions for achieving morally relevant human control over actions mediated by the use of complex autonomous technological systems; that is, they have not yet clearly defined the conditions under which humans may maintain control and moral responsibility for actions *mediated by the use of robotic intelligent systems with high levels of autonomy*.

Finally, unlike much of the existing literature on the ethics of military robots, we take the design perspective seriously and indicate how moral considerations can be utilized as requirements for design of systems. More specifically, we demonstrate how one might design for *meaningful human control*, thereby extending the approach of value-sensitive design in ethics of technology to a new domain of cases.

The notion of “meaningful human control” is meant to capture three ideas. Firstly, simple human presence or “being in the loop” is not a sufficient condition for being in control of a (military) activity. It is not sufficient because one can be present and perfectly able to influence some parts of the system by causal intervention, while (a) not being able to influence other parts of the causal chains that could come to be seen as even more relevant from a moral point of view than the parts one can in fact influence, (b) not having enough information or options to influence the process, for instance, if the human task consists in “merely pushing a button in a reflex when a light goes on” (Horowitz and Scharre, 2015). Secondly, controlling in the sense of being in the position of making a substantive causal contribution to a (military) activity through one’s intentional actions might not be a sufficient condition for meaningful control either, for instance, if one does not have the psychological capacity to respond appropriately under the circumstances and/or they are not in the position to appreciate the real capabilities of the system they are interacting with. Thirdly and relatedly, whereas some forms of legal responsibility (tort liability, strict liability) require only that the agents have relatively simple forms of causal control over events, other forms of legal responsibility (typically criminal responsibility) usually require stricter control conditions of knowledge, intention, capacity, and opportunity and therefore no matter how strong the political will to keep some human responsible or accountable for the behavior of autonomous weapon systems, attributions of legal responsibility that are not grounded in the relevant control conditions, may turn out to be not only morally unfair but also difficult to enforce in tribunals (Saxon, 2016). Reduced control over autonomous weapon systems may lead to a so-called responsibility gap (Matthias, 2004; Sparrow, 2007; Santoro et al., 2008; Human Right Watch, 2015; Santoni de Sio and Di Nucci, 2016) or “accountability vacuum” (Heyns, 2013).

In line with these concerns, some proposals have been put forward for preserving meaningful human control and human responsibility over autonomous weapon systems. Roorda (2016) claims that meaningful human control over autonomous weapon systems can be preserved by respecting the current NATO *targeting procedures*, i.e., through a correct planning and deciding on the means to be used. In partial contrast with this, O’Connell and Marry (2014) and Asaro (2012) insist that the presence of a human operator who takes a “near-time decision” to initiate each individual attack is always necessary to maintain meaningful human control over autonomous weapon systems. As for responsibility, it has been suggested that in order to prevent undesirable accountability gaps, commanders should receive an appropriate training (Saxon, 2016) and programmers should be made aware of their moral responsibility (Leveringhaus, 2016).^6^ It may be the case that one or more of these conditions are necessary and/or sufficient in order to achieve meaningful human control. However, we think that in the absence of an adequate comprehensive account of what meaningful human control is, it is difficult to assess how meaningful human control can be designed for and achieved. Therefore, in the following sections, we sketch a new account of meaningful human control. Before we do so, we will briefly explain the nature of the relation between control and moral responsibility in the current philosophical debate.

## The Philosophical Landscape: Control and Moral Responsibility

The debate on moral responsibility focuses on the question as to whether and under which conditions humans are *in control of* and therefore *responsible for* their everyday actions. *Incompatibilists* believe that humans can be in control of and morally responsible for their actions if and only if they possess a special power to make decisions and carry out actions that escape the causal influence of genetic (neuro)biological, sociopsychological, and circumstantial factors. They are called incompatibilists because they deny the compatibility of causal explanations of human actions and human moral responsibility—causality and moral responsibility cannot be reconciled. Incompatibilists can be divided in two main groups, depending on which of the two incompatible notions they hold on to. Some are *libertarians* (van Inwagen, 1983; Kane, 1996; Hodgson, 2012): in line with a philosophical tradition that goes back at least to Immanuel Kant, they believe that humans possess a special kind of autonomy, a “contra-causal” power which gives them a special metaphysical status and makes them morally responsible for their actions in a sense in which no other creature is (or can be). Other incompatibilists are *free will skeptics*—they believe that humans are not autonomous in any special sense; that is, they do not possess any special power to escape the causal influences on their behavior and therefore, from a philosophical point of view, they are never morally responsible for their actions; human actions are, as it were, in no relevant way different than natural events. Skepticism on free will and moral responsibility may be grounded in causal determinism in general (Pereboom, 2001), in the pervasiveness of luck factors (Levy, 2011), in neurobiological reductionism (Cohen and Greene, 2004), or in the pervasiveness of unconscious psychological mechanisms (Caruso, 2012).

In contrast with both forms of incompatibilism, *compatibilists* believe that humans may be morally responsible for (some of) their actions even if they do not possess any special metaphysical power to escape the causal influences on their behavior. *Traditional compatibilism* of modern philosophers like Hobbes and Hume was grounded in a mechanistic and associationist view of human mind. According to traditional compatibilism in order for agents to be morally responsible their actions need to be free only in the sense of being the causal product of internal motivational factors—desires, intentions, traits of characters, values—as opposed to being the product of “external” forces, i.e., the product of physical or psychological coercion. *Present-day compatibilists* (Frankfurt, 1971; Dennett, 1984, 2004; Fischer and Ravizza, 1998), while also rejecting the idea of strong contra-causal free will as a necessary condition for moral responsibility, rely on a more complex view of human mind and action. They reject the idea of mental causation as being sufficient to ground moral responsibility—many mentally caused actions are not responsible, for instance, those carried out by seriously mentally disordered persons—and they therefore see the capacity for rational control on actions as key for moral responsibility.

Present-day compatibilism can arguably offer the basis for an elaboration of an account of meaningful human control over autonomous weapon systems. Unlike incompatibilist libertarians, compatibilists do not ground human moral responsibility in any special, supernatural, exclusively human contra-causal power, and therefore they are not committed to the claim that *any* delegation of decision-making to non-human agents amounts *per se* to a disappearance of human moral responsibility over decisions and actions.^7^ Unlike free will skeptics however, compatibilists do recognize that there is a difference between human actions and other natural events, and they thus claim that human agents can be legitimately seen as morally responsible for at least some of their actions. Finally, unlike traditional compatibilists, present-day compatibilists try to offer a more complex view of the kind of the control over actions required for moral responsibility, one that does not simply coincide with the causal power to bring about effects in the world through one’s desires and intentional actions.

While we are not taking any particular position in the general debate on (compatibilist) theory of moral responsibility, in the following section we focus on one very influential compatibilist theory of human control over everyday action: Fischer and Ravizza’s (1998) theory of “guidance control”; we do so as we think that Fischer and Ravizza’s theory of control—whatever its merits as a theory of responsibility—is a very promising starting point for an account of meaningful human control over autonomous (weapon) systems. In the following sections, we apply a version of Fischer and Ravizza’s theory—enriched with insights from Nozick’s theory of knowledge—to socio-technical systems, of which autonomous robotic systems are an example. The ideas developed here apply equally to automated decision-making on the basis of advanced AI working *via* Machine Learning in a data environment.

## Conditions for “Guidance Control”

According to Fischer and Ravizza (1998), in order to be morally responsible for an action X a person should possess “guidance control” over that action. Guidance control is realized when two conditions are met: the decisional mechanism leading up to X should be (1) “moderately reason-responsive” and (2) the decisional mechanism should be “the agent’s own.”

The first condition of the reason-responsiveness of the decisional mechanism requires that the agent must act according to a decisional mechanism that in the presence of strong reasons to act (or to not act) can recognize these reasons and bring himself to (not) perform that action in a sufficiently broad range of circumstances.

The requirement of reason-responsiveness of the decisional mechanism marks the difference between morally responsible actors and actors acting under excusing factors such as (non-culpably) being under the influence of potent drugs, direct manipulation of the brain, behavior attributable to a significant brain lesion or a neurological disorder, phobias, drug addiction, and coercive threats (Fischer and Ravizza, 1998: 35–6). In all of these cases, the person’s decisional mechanism is by-passed or not responsive enough to moral reasons: it would (and it does) lead to actions even in the presence of strong contrary moral reasons recognized by the agents, or it fails to lead to certain actions even in the presence of strong moral reasons to act. The latter could be the case of an agoraphobic who does not venture onto the street to help someone who could be rescued without serious risk. Although the person acknowledges the validity of the moral reasons in favor of helping the person in the street, his phobia makes extremely difficult for him to do what he recognizes as the right thing to do. Two important clarifications are in order here. Firstly, the reference to possible alternative scenarios does not imply that the agent is able to bring about such scenarios; it only serves to ascertain that the *actual* mechanism has “some actually operative dispositional feature” (52–53). Secondly and relatedly, the focus of the theory is not on the circumstances or motivational factors that the agents can manipulate, but rather on the characteristics of processes or “mechanisms” leading to action, on their sensitivity, flexibility or lack thereof (38).

According to Fischer and Ravizza’s second condition for guidance control, in order to be morally responsible for their actions, the decisional mechanism should also be an integral part of who the agent is. This means that the agent must have “taken responsibility” for that mechanism by which she decides. “Taking responsibility” for one’s decisional mechanism requires that (a) the agent sees that her decisions have certain effects in the world; (b) the agent sees that others may have moral reactions toward her because of how she affects the world; and (c) the views specified in the first two conditions are based on the agent’s evidence in an appropriate way (Fischer and Ravizza, 1998: 207–239; Fischer, 2004: 146). If you consciously decide to settle an important matter by the toss of a coin, you are aware that you have decided to rely on what you perfectly know to be a random decision-making system and you have to accept that others will hold you accountable for the outcome of the choice (you cannot blame the system).

This second condition for guidance control, which may be called the “ownership” condition, marks the difference between morally responsible actions and behavior resulting from a reason-responsive mechanism that the agent has reasons *not* to consider as her own, like in cases of psychological manipulation, subliminal persuasion, strong nudging, strong entrapment, brainwashing, and indoctrination.

## Meaningful Human Control: Tracking and Tracing Conditions

Fischer and Ravizza’s theory of guidance control presents the conditions for human agents being morally responsible for their everyday actions, based on the features of the decisional mechanism leading to those actions, as well as the relation between the agent and the decision-making mechanism.

Fischer and Ravizza primarily consider intra-personal decision mechanisms, i.e., the mechanisms of an individual human mind or brain; however, an influential part of present-day cognitive science and philosophy of mind claim that human decisions and actions and even human personality traits are not represented in any brain mechanism; these accounts describe the human mind as “extended, embedded, and embodied” (Clark and Chalmers, 1998; Alfano, 2013). Accordingly, also the idea human control over actions may and should be applied on a larger scale to include artifacts and engineering systems (Di Nucci and Santoni de Sio, 2014); in fact, if we consider autonomous (weapon) systems as part of the decision-making mechanisms through which human agents carry out actions in the world, then Fischer and Ravizza’s conditions for guidance control on everyday actions may provide the basis for an account of meaningful human control over (military) actions mediated by autonomous (weapon) systems. In what follows, we offer an outline of such an account of meaningful human control by elaborating, in turn, on each of the two conditions for guidance control presented by Fischer and Ravizza. Fischer and Ravizza’s first condition will be enriched with some insights from Robert Nozick’s theory of knowledge.

### Tracking

Fischer and Ravizza’s first condition for guidance control, the *reason-responsiveness* condition, requires that the agent’s decisional mechanism is sensitive and responsive to a sufficient variety of moral input, i.e., that the decision-making mechanism can adapt the behavior of the system to the relevant moral features of the circumstances. These comprise both mental states of human agents and features of the external world. What seems required is that behavior of the system (the human operators, and the complex system, including interfaces that support decision-making) covary with moral reasons of a human agent for carrying out X or omitting X. We propose to redefine Fischer and Ravizza’s idea of reason-responsiveness in the terms of what Robert Nozick’s calls “tracking,” and we define a first condition of *meaningful human control* in terms of a so-called *tracking* relation between human moral capacities to respond to relevant moral reasons and (military) systems actions.^8^

The idea of tracking was introduced by Nozick (1981) in his account of knowledge. Nozick describes four conditions for how a person, S, can have knowledge of a proposition, P. In doing so he argues against the so-called traditional tri-partite accounts of knowledge and some of their successors.

The traditional accounts equate knowledge with true, justified belief. But they are open to so-called Gettier counterexamples which present believable cases where the conditions are all satisfied, i.e., where we have true justified belief, but we still do not think there is knowledge (Gettier, 1963). An example updated from Dancy (1985) construes a case. We are watching a Wimbledon final between Federer and Nadal, where Federer is about to beat Nadal. We go and get a coffee in the kitchen, and come back to watch the rest of the game. We see that Federer is serving a match point, and we switch off the television and conclude that Federer is this year’s Wimbledon winner. Unbeknownst to us it started to rain at center court while we were in the kitchen and the BBC started to broadcast last year’s final Nadal–Federer in which Federer also beats Nadal. After we switched off, the game was resumed and Federer indeed beat Nadal. Here, we have a situation where our belief that Federer is the Wimbledon’s champion is true and justified, but we do not say however that we *know* that Federer is the Wimbledon champion. In order for a subject S (in our example: myself) to know a proposition P (“Federer is the Wimbledon champion), Nozick claims that the following conditions must be met:

(i) P is true (Federer is the Wimbledon champion).
(ii) S believes that P (I believe that Federer is the Wimbledon champion).
(iii) If it were not the case that P, then S would not believe that P (if Federer was not the Wimbledon champion, I would not believe that he is the Wimbledon champion).
(iv) If it were the case that P, then S would believe that P (if Federer was the Wimbledon’s champion, then I would believe that he is the Wimbledon’s champion).

In the example above condition (iii) is not satisfied, because if Federer had eventually lost the final, I would still believe that he won. Nozick’s definition is known as a truth-tracking account of knowledge, because mental states in human minds should *track* the states of affairs in the world for them to constitute knowledge, in pretty much the same way a so-called tracker fund at the stock market just follows what the stock exchange is doing and the mercury column in a thermometer tracks the temperature in the room in a lawlike manner.

In his explication of the tracking relation, Nozick makes use of the so-called subjunctive conditional (“if it were the case…”) a non-truth-functional logical operator that differs from the standard material conditional “if–then” to capture the required robustness. If states of affairs in the world were different, the agent or the tool or the method would still respond in the right way. So, let us say that a military agent A uses a mechanism, system, or method M, which mediates between her and the world, being a method to acquire beliefs whether something is the case or not, e.g., whether children are present at the target (where P symbolizes that children are present).

The tracking element in Nozick’s account now implies:

(i) If P were not the case (i.e., children would not be present), and A were to use method M to arrive at a belief whether or not P, then M would not believe that children are present.
(ii) If P were the case (i.e., if children were present) and A were to use system or method M to arrive at a belief whether or not P, then M would believe that children were present.

This account of knowledge spells out in greater detail what it means to claim that a system is a reliable device or method to get to know the world as it is. By characterizing tracking in terms of subjunctive conditionals, it is not implied that those forms of AI and machine-learning tools that are probabilistic in nature would not be *per se* eligible candidates for satisfying the tracking relations. It just means that whatever its nature or functioning, the system should be able to respond to the world’s features in a satisfactory way; this also means that, in practice, we may need to set a reasonable threshold for judging “how much” reliability in the system responding to the relevant features of the world qualifies as “tracking” for a particular purpose.

In our case, the system actually used should exhibit a dual-tracking relation. The system environment should not only make it the case that the human agent’s belief states track the relevant states of affairs in the world, when the system is used as a decisional method as specified above; the system should also track the (relevant) moral reasons of the relevant agents deploying the method and it should effectively implement them.^9^ If the moral reasons of the human agents were different (there were no children after all, but instead young men) in a morally relevant sense (they were carrying explosives, and so they could be in principle considered as a legitimate target of a military attack), the mechanism would accommodate them so as to justify a change of plans, then the system would change its behavior accordingly. If on the other hand *the world* were different in a morally relevant sense, the mechanism would also track those altered states of affairs and represent them accordingly.^10^ If that would lead to an update or significant change of the moral reasons of the agent, the mechanism would then of course need to accommodate that change.

Following Fischer and Ravizza conditions of reason-responsiveness and Nozick’s concept of “tracking,” we may thus identify a

*First necessary condition of meaningful human control*. In order to be under meaningful human control, a decision-making system should demonstrably and verifiably be *responsive* to the *human* moral reasons relevant in the circumstances—no matter how many system levels, models, software, or devices of whatever nature separate a human being from the ultimate effects in the world, some of which may be lethal. That is, decision-making systems should *track* (relevant) human moral reasons.

Systems that do not display such a twofold epistemic and moral tracking reason-responsiveness, no matter how efficient they may be in performing specific tasks and even in achieving some broad and morally worthy human goals, would not qualify as being under meaningful human control.^11^ They would be like human actions carried out under the influence of potent drugs, phobias, or neurological disorders: a behavior that is clearly under the *causal control* of the human mind, but not under the right kind of *rational control* that grounds moral responsibility.

There are many cases where intelligent and highly autonomous systems have misrepresented the relevant states of affairs and as a result have not been able to behave in accordance with to the relevant human reasons. Many airplane crashes are the result of erroneous sensor data and inaccurate positioning or way point data. Human moral reasoning about the world in these cases is bound to lead to flawed outcomes. A famous example would be the Goalkeeper system used by the British Navy in the Falkland war, which misclassified an incoming Exocet rocket as friendly (i.e., French), when it was in fact deployed by the Argentinian enemy. In this case, the system demonstrated its inability to track some morally relevant state of affairs: a rocket being “friendly” as opposed to just being of the kind usually used by allies; and because of that it was also unable to track the relevant moral reasons of the human commanders: targeting enemy rockets rather than just targeting rockets with certain material features. More recently, the use of machine-learning systems has allegedly led to misclassification of enemy and friendly tanks because the training set had many images of enemy tanks with clouds and many of friendly tanks with cloudless skies or tracking higher or lower resolution (Yudowsky, 2006). These systems were also tracking irrelevant properties in the training set.

The importance of tracking for human control may be further clarified by looking at the following example. A machine-learning algorithm learns in supervised learning how to make a distinction between photos of wolves and photos of huskies. It eventually learns how to do that and unfailingly classifies new pictures as either huskies or wolves. It turns out, however, that the system just looks at presence of snow in the background, since the majority of wolves’ picture had snow in the background, whereas the husky photos did not. The system was giving good results by tracking the wrong property. If it *were* presented with a husky in the snow, it would have classified it as a wolf, since it was tracking the color and texture of the background, not the features of the animal itself (Ribeiro et al., 2016). In order for such a recognition system *to track the relevant properties* of huskies and wolves, the following conditions must apply:

(i) When presented with a wolf, the System classifies object as “Wolf”
(ii) If it were to be presented (in a great variety of possible worlds, e.g., snow and green vegetation) with a non-wolf (e.g., husky), then it would not classify the object as “Wolf” but as “Husky” or “non-Wolf.”
(iii) If it were to be presented (in a great variety of possible worlds) with a wolf, then it would classify it as “Wolf.”^12^

Our definition of tracking does not specify who are the agents whose reasons should be tracked in order for the system to be under meaningful human control. The only constraint explicitly contained by the definition is that these should be *human* agents. This means two things: first, a system may be under meaningful human control even if it does not track the reasons of the operator or the deployer under all circumstances, provided that it sufficiently tracks the relevant reasons of some other human agents along the chain: designers, programmers, legislators, policy-makers, etc. Second, a system may be under meaningful human control and pursuing bad or wrong goals or values. Meaningful human control is a necessary but not sufficient condition for a system to be morally or societally good.^13^

However, this does not mean that our definition of the tracking condition is morally neutral. In fact, insofar as it requires the system to respond to the human moral reasons *relevant* in the circumstances, it contains an important normative element; establishing whose moral reasons and which moral reasons are relevant in given circumstances means establishing which normative principle, norms, values a given system is supposed to follow or reflect. So, even by agreeing on tracking being a necessary condition for meaningful human control, it is still possible to disagree on whether tracking is realized under specific circumstances (due to a normative disagreement on the norms and values to be complied with or realized by a system under specific circumstances).^14^

### Tracing

Fischer and Ravizza’s second condition for guidance control, the *ownership* condition, may be characterized in terms of a *tracing* condition. The idea of tracing is often used by moral responsibility theorists and according to Manuel Vargas tracing “is one of a few things to which nearly all parties in the debate about free will [and moral responsibility] appeal to with equal enthusiasm” (Vargas, 2005 quoted by Timpe, 2011). The concept of tracing tries to capture the basic intuition that a human agent may be responsible for an outcome even if she does not satisfy the conditions for responsibility *in situ* at the time of her action, provided that she was responsible at an earlier time for finding herself later in the position of not satisfying those conditions. A typical example would be the drunk driver who causes a serious accident while in a state of mental incapacitation (and thus not satisfying the condition for responsibility at that time), but is responsible for choosing to drink at an earlier moment while knowing that she would drive and that her drunken driving may cause a serious accident. We therefore say that the driver’s responsibility for causing the accident *traces* back to the moment of her choice to drink (and/or to drive while drunk). Likewise, the driver’s responsibility for causing the accident could, in another scenario, be eliminated by tracing back to the moment that someone else tampered with the brakes of his car or unbeknownst to him put a drug in his tea. A similar conclusion is drawn by Kamm (2007) with respect to the case of Jim, the captain, and some prisoners originally introduced by Smart and Williams (1973) (p. 93–4). Jim is presented with a dilemmatic choice by the captain to select a prisoner to be shot. If he refuses then all prisoners will be shot. According to Kamm, the threat originates with (and therefore the responsibility traces back to) the captain, not with Jim. The captain is the choice architect, who creates Jim’s dilemma in which there are only tragic choices to be made by Jim. Fischer and Ravizza, Vargas, and other moral responsibility theorists are mainly concerned with scenarios where (a) only one individual agent is considered and (b) tracing applies to relations between different actions and mental states of one and the same human agent: in the drunken-driving example, between the agent’s dangerous driving and her drinking before getting into the car.

The challenge for an account of meaningful human control over autonomous systems is twofold. We need to extend the tracing condition to scenarios where there (a) is more than one human agent and (b) are intelligent non-human (sub)systems involved in the realization of the outcome. This challenge is not completely new. van den Hoven (1998) and Franssen (2015) have discussed cases of the distribution of responsibility between operators and designers regarding the use of intelligent systems (e.g., pilot, navigation systems, and system designers). Modern airplanes have complex semi autonomous systems onboard, including Collision Avoidance Systems (CAS), that make them swerve into safety and allows them to rapidly coordinate with other airplanes that are dangerously close by. The default policy is to defer to CAS in split second emergency situations. The pilot can, however, override the system. There are policies in place for the warranted overruling of CAS, for example when the pilot of the other plane that is on collision course obviously overrides CAS. According to Van den Hoven the pilot is supposed to check his work environment before he enters into it and is supposed to establish whether it will allow him to do what he ought to do. Or to have it inspected by others on his behalf. If he fails to do so when he can and finds himself later “locked-in” in a system’s environment that does not allow him to discharge his obligation to avoid a collision, then he may be held morally responsible for a negative outcome as his responsibility can be traced back to his failing at an earlier moment to do a proper check.

However, in doing his checks, the pilot is relying on numerous others who have shouldered a part of that burden in the design and production history of the system he is operating. It is unavoidable that the pilot makes certain assumptions and relies on the expertise and good will of numerous others: engineers, inspectors, mechanics, administrative staff, etc. This means that sometimes the pilot may not be responsible for an accident: it may well be the case that specific others in the etiology of the system design, production, and maintenance have not discharged their second-order responsibility for the first-order responsibility of the operator *in situ*. In case it turns out that conditions for operator or user first-order moral responsibility are not satisfied as a result of no fault of the operator, then the responsibility for the accident may *trace* back to other agents upstream, or to the designers of the system, who fail to exemplify proper understanding of the system.

In general, users and operators who are somehow related to the loop (in or on, or half in) or otherwise involved in the deployment of the system have a so-called meta-task responsibility, i.e. an obligation to check whether the system is responsive to the dynamic moral reasons of relevant moral agents that obtain and that apply to them *in situ* (van den Hoven, 1998). They have an obligation to check whether the system allows them to do what they ought to do *in situ*. One interesting implication of this analysis is that the designers, producers, and architects of these elaborate systems have an obligation to design the system in such a way that this type of inquiry by users is not made impossible or unduly difficult. They could be said to have an obligation to facilitate that kind of inquiry.

Based on the analysis of this section, we thus propose to define a second condition of *meaningful human control* over autonomous systems in terms of a *tracing relation* between the decision-making system and the technical and moral understanding of some relevant humans involved in the design and deployment of the system. In order to do so we rely on the general idea of tracing and on Fischer and Ravizza’s second condition for guidance control, the *ownership* condition, which requires the agent has to properly understand and endorse the mechanism of moral decision-making leading to her action. In this way we formulate a

*Second necessary condition of meaningful human control*: in order for a system to be under meaningful human control, its actions/states should be traceable to a proper moral understanding on the part of one or more relevant human persons who design or interact with the system, meaning that there is at least one human agent in the design history or use context involved in designing, programming, operating and deploying the autonomous system who (a) understands or is in the position to understand the capabilities of the system and the possible effects in the world of the its use; (b) understands or is in the position to understand that others may have legitimate moral reactions toward them because of how the system affects the world and the role they occupy.

Systems whose actions and states are not traceable to relevant understanding and endorsing by some human person—be they a designer, a controller, a user, etc.—no matter how intelligent and reason-responsive they may be, are not under meaningful *human* control. They would be like human actions carried out under psychological manipulation, subliminal persuasion, brainwashing, and indoctrination; here, the agent’s behavior is clearly responsive to *someone*’*s* reasons, but not to the agent’s reasons.

## Meaningful Human Control Over Autonomous Weapon Systems: Implications of Tracking and Tracing

Based on this account of meaningful human control, we are now in the position to assess the merits of the existing position in the ethical debate on autonomous weapon systems. In general, critics of autonomous weapon systems (e.g., Peter Asaro and Noel Sharkey) seem to be right in stressing that current robotic systems are not able to honor the dual-tracking relation. First, they are likely to fail in tracking the relevant reasons of the human military personnel behind them; in particular, they cannot track the reasoning required by international law (including being guided by considerations of necessity, discrimination, and proportionality). Secondly, they are not as flexible as to properly adjust their behavior to the many morally relevant features of the environment in which they operate. If they would be able to distinguish between civilians and combatants, they need also to be able to distinguish between *civilians* and *civilians apparently involved in armed resistance* in unstructured and dynamic environments like the ones of present-day battlefields. Therefore, if autonomous robotics systems were given the possibility to take the decision to initiate an attack without human supervision in an unstructured environment that would *not* be under meaningful human control.

However, in contrast with Asaro and others’ statement that the presence of a human operator who takes a “near-time decision” to initiate each individual attack is a necessary condition to achieve meaningful human control over autonomous weapon systems, Roorda (2016) has suggested that autonomous weapon systems may remain under sufficient control even if no human operator is involved in the “engagement” (attack) stage of the military operation, provided that military commanders in charge of the decision to deploy these weapon systems have followed an appropriate targeting procedure, for instance, those of NATO. If these targeting procedures are properly followed, so the reasoning goes, autonomous weapon systems will be (lawfully) used only in circumstances where they can behave according to the human commanders’ intentions and reasons. If they are deployed outside these circumstances, the moral and legal responsibility for their behavior will clearly be traceable to the conscious and culpable decision of the commander. In both cases, the behavior of the autonomous system will be under meaningful human control, and there will not be any accountability gap.

Roorda’s argument rightly points to one key general aspect of meaningful human control as analyzed in this paper: being in control does not necessarily require the act of direct controlling from a position that is contiguous in space and time or is a proximate cause, as control in a morally relevant sense allows for technological mediation and separation of the human agent and the relevant moral effects of the acts that he is involved in. Moreover, we concur with the general idea that social and legal practices also contribute to create people’s moral identity and to make them legitimate targets of moral and legal responsibility attributions. In fact, whether an agent can legitimately be seen as in control of a certain outcome, and thus be legitimately held responsible for that outcome in retrospect, also depends on the normative position occupied by that person within a recognized social or legal architecture of duties and responsibilities. In this perspective, the presence of an appropriate social and legal system of rules is also a necessary component of a socio-technical system that prevents responsibility gaps and achieves meaningful human control over autonomous weapon systems. However, Roorda’s argument is unsatisfactory insofar as it seems to assume that current social and legal practices will be enough to maintain any (future) autonomous weapon system under meaningful human control.

In order to see why this assumption is unwarranted, we have to consider our tracing condition for meaningful human control: every action of a decision-making system should be traceable to a proper technical *and* moral understanding on the part of at least one human among those who design and deploy the system, meaning that at least one human agent is at the same time: (a) in the position to understand the capabilities of the system and the possible effects in the world of its use and (b) in the position to understand that others may have legitimate moral reactions toward them because of how the system affects the world. It is doubtful whether under the current socio-technical circumstances military commanders may comply with any of the two subconditions. As for (a), the former prosecutor at the International Criminal Tribunal for the former Yugoslavia Dan Saxon recently remarked that the introduction of autonomous weapon systems is likely to significantly “increase the demands on the General’s already taxed mental capacity.” Prior to any deployment of such technology, the General must consider: (1) the range, accuracy, and explosive power of the autonomous weaponry to be directed at the enemy vis-à-vis human-operated weaponry and the possible presence of civilians in the area; (2) the autonomous weaponry’s ability to comply with International Humanitarian Law in the particular battlespace; (3) whether the mission or the expected circumstances of the battlespace may require the exercise of increased levels of human supervision and control over the robotic weaponry; (4) whether the General and/or her staff will have the capacity to deactivate the autonomous drone immediately should conditions require it; (5) the robustness of the software that operates the artificial intelligence of the autonomous drone, in particular whether enemy forces may have the ability to tamper with and/or take control over the autonomous drone(s); and (6) the level of training—technical, operational, and with respect to the laws of war—of the human “operators” or monitors of the autonomous weapon systems (if any) (Saxon, 2016). Given the complexity and difficulty of this task, we cannot assume that just because they are required to do so by the official procedures, commanders will in practice be able to properly assess all these variables. In order for the system to remain under meaningful human control we need thus to ensure that military commanders have a sound understanding of the function, capabilities, and limitations of the autonomous weapon technologies available to them (ibid). We also have to be reminded that the military advantage provided by increasing speed in acquisition and transmission of information and reaction may influence decisions about acceptable levels of human judgment and permissible levels of autonomy (ibid).

Similar considerations apply to the part (b) of the tracing condition above: commanders should understand that others may have legitimate moral reactions toward them because of how the system affects the world, that is, they should realize that they are responsible for what the system does. It has been argued that the tendency for human beings increasingly to depend on computer systems for their decision-making can lead to a reduced sense of responsibility for the consequences of those decisions (Cummings, 2006; Coeckelbergh, 2013; Saxon, 2016). In other words, no matter how strict the legal obligation is that is imposed on commanders to take responsibility for the behavior of autonomous systems, the system may not be under meaningful human control if commanders do not perceive the ownership of these actions, due to a lack of sufficient training and experience with the use of these systems.

So, tracing as we defined it is not only meant, as it were, to help finding someone to blame after an accident has occurred; tracing is much more than that. Tracing is an essential component of meaningful human control over a system, because it requires that there always are individual humans along the chain who are capable and motivated to take active steps to prevent unwanted outcomes to occur in the first place.

Two further implications of our analysis are the following. First, systems that are generally unable to track some relevant moral reasons may still be under meaningful human control in a morally relevant sense if they track the relevant moral reasons of the relevant agents who deploy them. That means that meaningful human control is not a sufficient condition for morally appropriate behavior of a robotic systems. Consider, for instance, an autonomous weapon system that is unable to comply with the Laws of Armed Conflict and is used to perform an unlawful attack by a human commander who is perfectly aware of this inability but decided to use the system anyway to gain military advantage, which she eventually did. In this case, we argue not only the tracing, but also the tracking condition is satisfied, because though the system is clearly not responsive to some important moral reasons and is not responsive to many relevant features of the environment in which it operates, still it is responsive to the (wrong) relevant moral reasons of the relevant agent who deploys it and it is sensitive to those features of the environment which that agent wants it to be responsive to, in order to achieve her strategic goals. In fact, this would arguably be an unlawful but deliberate attack, for which the military commander would clearly be morally and legally culpable (Saxon, 2016).

Second, autonomous weapon systems may be outside of meaningful human control even if their capabilities and limits are well known to their human creator and there is no intention to misuse them on the part of the military personnel deploying them. According to our analysis, the system may still be out of meaningful human control if there is no individual human agent who is in the position to appreciate the limits in the capabilities of the machine while *at the same time* being aware that the machine’s behavior will be attributed to them; for instance, if the programmers are vividly aware of the limited capacities of the machines but do not feel responsible for their use, because they assume that military commanders will be able to discharge their duty to take the morally relevant decisions about the use of the system; and the military commanders are aware that it is their responsibility to take strategic decisions, but at the same time overestimate the capacity of the systems, due to insufficient training or experience in their use. Here, there is arguably a responsibility gap as no agent satisfies the tracing condition.

In conclusion, whereas our philosophical analysis offers support to the political concerns of critics of autonomous weapon systems, it also leaves open the conceptual possibility that future weapon systems with a high level of autonomy may remain under meaningful human control, provided that a series of technical and institutional advancements are realized, and their use is properly constrained to the right kind of operations.^15^

We think that the concept of meaningful human control could be applied beyond the domain of military robots. Therefore, before concluding, in the next final section we start exploring the implications of our account of meaningful human control for the design and use of non-military autonomous systems.

## The Broader Picture: Meaningful Human Control and Responsible Innovation in Robotics

Even though the concept of meaningful human control has emerged and has so far almost exclusively been used in the political discussions on the ban of fully autonomous weapon systems,^16^ we think that it can play an even broader role: it can be one of the central notions of thinking about Responsible Innovation in robotics and AI.

We believe that human control and accountability are important values to protect in all activities where basic human rights like life and physical integrity (as well as freedom and privacy) are at stake. After all, transport accidents, healthcare practices, and abuse of personal data may affect people’s life as much as military operations do. In line with this program, in this last section we start brushing the first strokes of a *general* theory of *design for* meaningful human control over autonomous systems, by looking at automated driving systems as a first example. Future work will develop these ideas more systematically.

Responsible Innovation and Value-Sensitive Design research focuses on the need to embed and express the relevant values into the technical and socio-technical systems (Friedman and Kahn, 2003; van den Hoven, 2007, 2013). From this perspective, the question to be addressed is how to *design* technical and socio-technical systems which in accordance with the account of meaningful human control we have here presented.^17^ Based on our analysis of meaningful human control, we propose the following two general design guidelines, and we briefly show how these can be applied outside the military context, by looking at the case study of automated driving systems (aka “autonomous vehicles,” “self-driving cars,” “driverless cars”).

The first condition for meaningful human control which we have identified is that an autonomous system should be able to track the relevant human (moral) reasons (in a sufficient number of occasions). Correspondingly, this is also our first design guideline. One interesting aspect of this condition is that meaningful human control is context- and norm-dependent: whether a given system is or not under meaningful human control crucially depends on what should count as the *relevant* moral reasons, what qualify as a *sufficient* responsiveness to those reasons, and the reasons of *which agents* the system should track. This means that in order to design for meaningful human control, we first need to identify the relevant human agents, and the relevant moral reasons at stake in different scenarios, as well as the level of responsiveness to those reasons required under different circumstances. For instance, in relation to automated driving systems, it will be required that the system is able to always comply with *all* the rules of traffic as defined by the society *via* the public authority, and sometimes with *some* unwritten conventions which govern human interaction in the traffic, and which reflect some relevant interest of the road users (but possibly not with some idiosyncratic interpretation of these norms by individual drivers) (Santoni de Sio, 2016); unlike, for instance, military or healthcare assistive robots, the system may arguably remain under meaningful human control even if it is not able to comply at all with the laws of armed conflict or with the moral norms which govern the caregiver–patient relationships. This condition also entails that the required level of responsiveness to the same kind of moral reason may change with the context. For instance, an interactive service robotic system operating in a sensitive domain like healthcare should arguably be more responsive to the signals of distress of the human user than an interactive service robot operating in a commercial setting.

Another important design implication of the tracking condition is that meaningful human control can be achieved and enhanced not only by sharpening the responsiveness of the robotic system to the relevant moral reasons, but also by designing the environment in such a way as to reduce or eliminate the occasions of encountering morally challenging circumstances. In the case of an autonomous driving system, for instance, we may arguably maintain under meaningful human control a vehicle which is not able to safely interact with pedestrians and cyclists by designing the traffic infrastructure in such a way as to simply prevent the possibility of this interaction, for instance, by providing separate lanes for autonomous and traditional vehicles.

The second condition for meaningful human control requires that the behavior of an autonomous system is traceable to a proper moral understanding on the part of humans who design and deploy the system. This condition extends the scope of the design task to a third level in addition to the level of the design of the robot and that of the design of the environment: the design of social and institutional practices. Designing for satisfying the tracing condition means ensuring that different human agents along the chain are technically and psychologically capable of complying with their tasks and are well aware of their responsibility for the behavior of the autonomous system. The design challenge of realizing the tracing condition of meaningful human control is thus twofold (cfr. Santoni de Sio, 2016). Not only do we need to understand what the ideal distribution of tasks between humans and robots is, from a functional point of view; for instance, in a vehicle equipped with assisted cruise control, which driving operations should be delegated to the computer and which should remain with the human driver. We also need to engage in a social and psychological investigation to understand under which circumstances the human drivers are in practice able and motivated to do their part when requested; in the case of assistive cruise control, this means acquiring sociopsychological data to assess the reasonableness of the normative expectations attributed to the driver to perform certain tasks and supervise certain operations; it may also mean filling possible psychological gaps by an appropriate design of new systems of training and licensing for users. In this perspective, in order to implement the tracing condition, to enhance meaningful human control, and to reduce the risks of “responsibility gaps” we not only need—as it is often claimed—to design appropriate new normative systems, for instance, new legal rules for attributions of liability in the event of accidents involving autonomous systems. We also need to design educational and training systems to improve the understanding of the functioning of these systems and the risks and responsibilities associated with designing and operating them.

## Conclusion

Meaningful human control has played a key role in the recent ethical, political, and legal debate on the regulation of autonomous weapon systems. In this paper, we have presented a philosophical account of this concept, based on an elaboration and extension of the concept of “guidance control” proposed by Fischer and Ravizza in the debate on free will and moral responsibility, integrated by Nozick’s notion of tracking. Based on this analysis, we have realized two goals: we have given a more solid philosophical foundation to the ethical reflection on the deployment of autonomous systems in warfare and we have paved the way for a broader theory of meaningful human control over autonomous robotic systems in general.

## Author Contributions

JvdH first conceived of the present approach to meaningful human control. FSdS developed the concept and took the lead in the writing of the paper. JvdH wrote the section on tracking and part of the section on tracing. Both authors have done multiple integrations and revisions of the draft.

## Conflict of Interest Statement

The authors declare that the research was conducted in the absence of any commercial or financial relationships that could be construed as a potential conflict of interest. The reviewer, TB, declared a past coauthorship with one of the authors, FSdS, to the handling editor.

## Footnotes

^1^Many different definitions of autonomous weapon systems have been proposed. See Verdiesen (2017) for a critical review.

^2^Moreover, weapon systems with autonomous capabilities such as the PATRIOT missile system and the Goalkeeper ship defense system have been in use for some decades now; however, they arguably do not qualify as “fully autonomous” weapon systems insofar as they are embedded in a decision-making process where a human needs to take action in the clearance procedure to take out a target.

^3^The US military has recently presented a fully autonomous submarine: “Sea hunter” http://www.reuters.com/article/us-usa-military-robot-ship-idUSKCN0X42I4; and Lockheed Martin and the U.S. Air Force have demonstrated that a drone that can detect and respond to unexpected threats with no human intervention required http://www.airspacemag.com/daily-planet/no-pilot-no-problem-180963050/.

^4^http://www.unog.ch/80256EE600585943/(httpPages)/6CE049BE22EC75A2C1257C8D00513E26.

^5^https://www.hrw.org/news/2016/04/11/killer-robots-and-concept-meaningful-human-control.

^6^Roff and Moyes (2016) have recently sketched a general framework for understanding meaningful human control, which tries to combine these elements; however, their strategy is rather that of identifying “broad ‘key elements’” or “general principles cumulatively constituting meaningful human control” (p. 6), rather than giving a definition or sketching a philosophical theory of meaningful human control.

^7^This idea is often supporting the claim of autonomous weapon systems as *mala in se* (see Introduction).

^8^The use of the terms “tracking” here and “tracing” below follow the philosophical usage and does not exactly match the use in the military or in the transport domain.

^9^Which reasons of which agents the system should track cannot be establish *a priori*, and it should be determined on a case-by-case basis, looking at the system, the chain of command, and at the circumstances in which the system operates; more on this in Section “Meaningful Human Control over Autonomous Weapon Systems: Implications of Tracking and Tracing.”

^10^So meaningful human control doesn’t offer in itself any safeguard against evil human controllers; more on this in Section “Meaningful Human Control: Tracking and Tracing Conditions.”

^11^It has to be noticed that our account of meaningful human control is certainly demanding in that it requires the system to be able to track the moral and legal reasons relevant in a given context of action; however, it is less demanding than other accounts insofar as it does not require a system to have any general capacity for moral understanding. More on this below, in Section “Meaningful Human Control: Tracking and Tracing Conditions.”

^12^An additional complication for the tracking condition is its being sensitive to variations based on the epistemic conditions of the humans interacting with the technical system. A gauge in the cockpit that always indicates that there is 10% more fuel in the tank than in fact there is, and is known to have this positive bias by the crew does track the fuel levels to the old crew, but not to a new crew, who have not been informed of this bias.

^13^An example of a military system that satisfies the tracking condition while at the same time being used for illegal purposes is given in Section “Meaningful Human Control over Autonomous Weapon Systems: Implications of Tracking and Tracing.”

^14^For similar reasons, in Section “The Broader Picture: Meaningful Human Control and Responsible Innovation in Robotics,” we claim that tracking is also context-sensitive. We also provide an example of a potential normative disagreement about the relevant reasons to be tracked by an autonomous driving system, i.e. whether in order to be under meaningful human control the system should track only the written traffic rules or also some conventional norms usually followed in traffic or even some idiosyncratic reason of an individual driver.

^15^For suggestions in this direction, see Arkin (2009) and Kasher (2016). From this perspective, it may be argued that some defence systems maybe at the same time fully autonomous and under meaningful human control.

^16^See Asaro (2012) for a reconstruction of the recent story of the concept.

^17^The idea of designing for meaningful human control is a promising one also in the military context discussed above; in fact, even if *fully* autonomous weapon systems will be banned and never developed, more and more autonomous weapon systems will be developed, which will raise the issue of how to maintain them under meaningful human control.
